# Aspirin Versus LMWH for Thromboprophylaxis Following Hip or Knee Arthroplasty—Clinical Implications and Budget Impact

**DOI:** 10.1002/prp2.70147

**Published:** 2025-07-07

**Authors:** Eugen Javor, Andrej Belančić, Patrik Javor, Goran Hauser, Ivan Kruljac, Marko Skelin, Andrea Faour, Marko Lucijanić

**Affiliations:** ^1^ Solmed Clinic Zagreb Croatia; ^2^ Department of Basic and Clinical Pharmacology With Toxicology University of Rijeka, Faculty of Medicine Rijeka Croatia; ^3^ General Hospital Sisak Sisak Croatia; ^4^ Faculty of Medicine University of Rijeka Rijeka Croatia; ^5^ Department of Internal Medicine Clinical Hospital Centre Rijeka Rijeka Croatia; ^6^ Medical School of the Catholic University of Croatia Zagreb Croatia; ^7^ Pharmacy Department General Hospital Šibenik Šibenik Croatia; ^8^ Vancouver Coastal Health Vancouver British Columbia Canada; ^9^ School of Medicine University of Zagreb Zagreb Croatia; ^10^ Division of Hematology, Department of Internal Medicine Clinical Hospital Dubrava Zagreb Croatia

**Keywords:** aspirin, low‐molecular‐weight heparin, orthopedics, thromboprophylaxis

## Abstract

Venous thromboembolism (VTE) remains a significant concern for patients undergoing hip or knee arthroplasty, with a need to balance effective thromboprophylaxis and bleeding risk. We aimed to compare the efficacy, safety, and budget impact of aspirin versus low‐molecular‐weight heparin (LMWH) as sole thromboprophylactic agents initiated immediately postoperatively in this population. First, we conducted a systematic review of randomized controlled trials (RCTs) from Ovid MEDLINE, Embase, and Cochrane CENTRAL databases, assessing clinical outcomes and healthcare costs. Subsequently, a simplified budget impact analysis was performed using data from the largest identified and most recent RCT (CRISTAL trial) and its secondary analyses. Primary outcomes included symptomatic VTE, bleeding events, and reoperation rates. Through a systematic search, seven RCTs were considered to be eligible, with the CRISTAL trial providing the most compelling evidence. Aspirin was non‐inferior to LMWH for all‐cause mortality but was associated with a significantly higher symptomatic VTE rate (3,27% vs. 1,76%) and deep vein thrombosis (DVT), predominantly distal DVT. The budget impact analysis revealed that despite aspirin's lower per tablet cost, thromboprophylaxis with LMWH led to annual savings of $35,912,459 to $110,431,241 for U.S. healthcare stakeholders, and $17,075 to $56,450 for single hospitals performing 1000 arthroplasty procedures annually. To conclude, enoxaparin appears to offer superior clinical efficacy and cost‐effectiveness compared to aspirin for thromboprophylaxis following hip and knee arthroplasty. These findings support the preferential use of LMWH in this setting, while highlighting the need for further investigation into the clinical significance of aspirin's higher distal DVT and pulmonary embolism risk.

## Graphical abstract

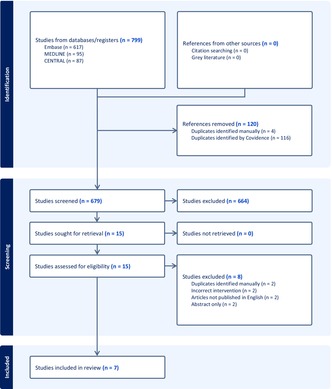

## 1 Introduction

Venous thromboembolism (VTE), which comprises deep vein thrombosis (DVT) and pulmonary embolism (PE), poses a significant risk during and after hospitalization for patients undergoing major orthopedic surgery, such as total or partial hip or knee arthroplasty. The incidence of symptomatic VTE is approximately 4%, with the greatest risk occurring within the first 7–14 days following surgery. Major bleeding events are reported at rates of 2%–4% [1]. One of the preferred options, besides low molecular‐weight heparin (LMWH), for thromboprophylaxis in hip or knee arthroplasty is direct oral anticoagulants (DOACs), particularly rivaroxaban and apixaban, due to more data to support their use in this setting. In the recent large meta‐analysis of 53 randomized clinical trials (RCTs) (44 371 participants) comparing the effects of direct factor Xa inhibitors to LMWH, there was no difference in all‐cause mortality, major bleeding, and serious hepatic adverse events between the interventions. Direct factor Xa inhibitors may have little to no effect on the reduction of major VTE and slightly reduce symptomatic VTE compared to LMWH, but in a subgroup analysis, rivaroxaban may have had more major bleeding events than LMWH. The meta‐analysis included the majority of participants undergoing total hip (23 RCTs) or knee (21 RCTs). Although compelling, these results are of low validity due to very uncertain, very low‐certainty, or low‐certainty evidence [2]. Some authors report that rivaroxaban may be even a cost‐effective alternative to enoxaparin for routine thromboprophylaxis following total knee or hip arthroplasty [3]. However, although DOACs are an interesting and important pharmacological approach for thromboprophylaxis, the focus of our research is aspirin, which is another attractive alternative to LMWH for thromboprophylaxis in hip or knee arthroplasty. Its use has increased in the past decade due to its low cost, perceived safety, and ease of administration [4, 5].

The Arthroplasty Society of Australia guidelines from 2023 recommend aspirin as an appropriate thromboprophylaxis option for patients at low or routine risk of VTE but deem it inappropriate for patients at high risk of VTE [6]. The American College of Chest Physicians (ACCP) guidelines from 2012 recommend aspirin as an appropriate thromboprophylactic agent post‐arthroplasty, though they prefer LMWH [7]. The American Academy of Orthopedic Surgeons (AAOS) Guidelines from 2009 recommend aspirin as one of the thromboprophylactic agents in patients at standard risk of both PE and standard or elevated risk of major bleeding [8]. The National Institute for Health and Care Excellence (NICE, UK) Guidelines from 2019 (revised) recommend LMWH for 10 days followed by aspirin (75 mg or 150 mg) for a further 28 days in patients undergoing hip arthroplasty and aspirin (75 mg or 150 mg) for 14 days as one of the preferred thromboprophylactic agents in patients undergoing knee arthroplasty [9]. In 2013, the EPCAT, a large multicenter RCT, demonstrated the non‐inferiority of aspirin compared to LMWH (dalteparin) for extended thromboprophylaxis in total hip arthroplasty (THA). All patients received an initial 10‐day course of dalteparin [5000 IU subcutaneously (s.c.)] thromboprophylaxis before being randomized to either 28 days of dalteparin or aspirin (81 mg/day orally) [10]. However, the role of aspirin versus LMWH as a sole thromboprophylactic agent used immediately postoperatively is still unclear. Therefore, we conducted a systematic review of RCTs that compared aspirin with LMWH administered immediately postoperatively. We have also performed a simplified budget impact analysis of aspirin versus LMWH monotherapy from a healthcare stakeholder or a hospital drug and therapeutics committee (DTC) perspective.

## 2 Materials

### 2.1 Search Strategy and Eligibility Criteria

**FIGURE 1:**
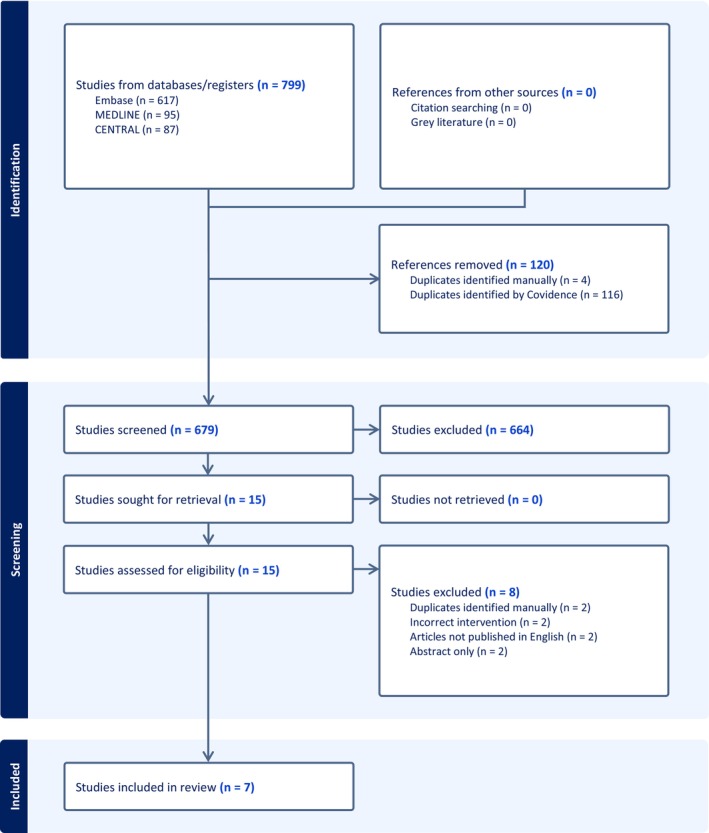
PRISMA flow diagram for a systematic review of RCTs that compared aspirin versus LMWH for thromboprophylaxis following hip or knee arthroplasty.

On November 7, 2024, Ovid MEDLINE, Embase, and Cochrane Central Register of Controlled Trials (CENTRAL) databases were searched for RCTs published in English, evaluating the efficacy and safety outcomes of aspirin versus LMWH in adults (≥ 18 years) undergoing hip and knee arthroplasty. The search terms included “aspirin,” “LMWH” or “enoxaparin” or “dalteparin” or “tinzaparin” or “nadroparin,” “orthopedic surgery” or “arthroplasty” or “knee arthroplasty” or “hip arthroplasty,” and “thromboembolism” or “thromboprophylaxis” or “thrombosis.” Secondary analyses of RCTs that fulfilled the inclusion criteria were included as well. RCTs that evaluated not only aspirin and LMWH groups (arms) but also other groups [e.g., direct oral anticoagulants (DOAC)] were included, as long as the RCT compared aspirin versus LMWH. RCTs that had a placebo as a comparator or had alternative forms of prophylaxis for the immediate postoperative period prior to aspirin prescription were excluded (Figure 1). Covidence was utilized to facilitate the screening and selection of studies for eligibility [11].

### 2.2 Data Collection and Extraction

For each of the included RCTs, we extracted the following parameters to assess the quality of evidence. The extracted data included trial type, year of publication, country of trial conduction, number of included patients, inclusion criteria (indication, type of surgery, trial population), exclusion criteria, intervention (dosing and duration of thromboprophylaxis), and primary and secondary outcomes. For the evaluation of efficacy and safety outcomes for aspirin and LMWH thromboprophylaxis, we extracted the following data (if available) from the included RCTs: all‐cause mortality, VTE, DVT [categorized into proximal DVT (PDVT), occurring above the knee, and distal DVT (DDVT), occurring below the knee], PE, major bleeding events, readmission, reoperation, and wound complications.

For the budget impact analysis, only multicenter RCTs evaluating hip and knee arthroplasty procedures were included. Single‐center RCTs deemed to provide less valuable evidence were excluded from the budget impact analysis. The following variables from the RCTs were used for the analysis: all‐cause mortality, VTE (comprising all efficacy outcomes), bleeding events, reoperation procedures, and readmissions. We chose to present cost projections in U.S. dollars ($, USD). THA and total knee arthroplasty (TKA) comprise the majority of all hip and knee arthroplasty procedures, and their yearly burden projections have changed over time. Earlier models projected exponential growth in THA and TKA procedures (primary and revision) [12]. However, the latest available model suggests linear growth in THA and TKA procedures (primary). Using linear regression modeling, the authors projected that the number of THA and TKA procedures performed by 2030 in the United States would be 635 000 and 1 257 800, respectively [13]. The projections from 2030 serve as a practical benchmark for estimating the future burden. Therefore, we decided to use these more conservative (linear growth) estimates and numbers for 2030 to project the annual burden of THA and TKA in the United States. We have also hypothesized a total of 1000 THA and TKA procedures jointly, in a distribution replicating annual THA and TKA burden in the United States (34% vs. 66%) [13] for a single hospital DTC perspective analysis.

All‐cause healthcare costs per patient with VTE vary between $18 000 to $23000 [14]. We decided to use the highest value of $23000 per VTE event during the first year to approximate the worst‐case scenario and account for inflation from the time of publication (2016) to the present (2025). The economic burden of bleeding and transfusions ranges from $9824 to $29296, depending on the surgery and the hemostatic agent used [15]. Bleeding events in orthopedic and spine surgeries tend to occur less frequently and are generally less costly than in cardiovascular/thoracic surgeries. Therefore, we opted to use the lowest value of $9824 for these calculations. For drug pricing, we used the Average Sales Price (ASP) for aspirin 81 mg ($0,10) and 100 mg ($0,12) tablets and generic (the lowest price) enoxaparin 30 mg ($12,80) and 40 mg ($18,00) [16]. The cost of revision TKA in high‐income countries varies, depending on the procedure type (septic or aseptic) and whether a re‐revision is involved. A two‐stage septic revision with re‐revision ranges from $66629 to $81938, one‐ or two‐stage septic revision without re‐revision ranges from $24027 to $38109, while aseptic revision without re‐revision ranges from $13910 to $29213 [17]. We decided to use the middle‐priced revision (one‐ or two‐stage septic revision without re‐revision) with the highest value of $38109, due to the inflation rate from the time of publication (2021) to the current year (2025). The cost of revision THA also varies. Direct costs are greatest for a two‐stage exchange ($37642) while lowest for a linear revision ($8979). Septic revisions, as expected, are more costly ($17696) than aseptic revisions ($11204) [18]. We decided to use the cost of a mid‐range septic revision ($17696) for project calculations.

The duration of thromboprophylaxis will be used from each included RCT and multiplied by the drug price to determine therapy costs. To determine the number of VTE, bleeding events, and reoperation procedures, the percentage of these outcomes in the included RCTs will be multiplied by the yearly incidence of projected procedures (hip and knee arthroplasty procedures) in the United States. To compare aspirin and LMWH thromboprophylaxis approaches, all projected costs for each approach will be summarized (Table 3). Data were extracted into a predesigned Microsoft Excel (ver. 2311, Microsoft, Redmond, WA, USA). The summary statistics and the economic evaluations were performed using Microsoft Excel.

## 3 Results

### 3.1 Clinical Implications

The search of databases yielded 799 articles (Ovid MEDLINE = 95 results, Embase = 617 results, CENTRAL = 87 results). Following the removal of duplicates, 679 articles were screened based on title and abstract, and 15 articles were deemed potentially relevant and underwent full‐text review. Two authors (EJ and AB) independently screened the articles. Any disagreements or differences in the article selection between the two authors were resolved through consensus after rechecking the source data. A total of seven RCTs [15, 16, 17, 18, 19, 20, 21] were included in the final analysis (Table 1), and the PRISMA flow diagram is presented in Figure 1.

**TABLE 1:** Overview of characteristics and methodology of eligible RCTs that compared aspirin versus LMWH for thromboprophylaxis following hip or knee arthroplasty.

*N*	Trial	Trial type	Country	Number of patients	Inclusion criteria	Exclusion criteria	Intervention	Primary outcome	Secondary/other outcome
1	Bukhari et al., 2023	Quasi‐randomized controlled trial, single center	Pakistan	Aspirin (*n* = 37), Enoxaparin (*n* = 37)	THA (cemented and non‐cemented) or hemiarthroplasty, aged 18–70 years	Bilateral procedures, hypersensitivity to either LMWH or aspirin, long‐term anticoagulation therapy, unfit for surgery (ASA III and above), family unwilling to give informed consent, and revision hip arthroplasty	Aspirin (75 mg/day) or Enoxaparin 0.5 mg/kg for 14 days	VTE within 14 days of surgery	
2	CRISTAL Study Group 2022	Registry‐nested, crossover cluster‐randomized controlled trial, multicenter	Australia	Aspirin (*n* = 5416), Enoxaparin (*n* = 3787)	Primary elective total unilateral or bilateral THA or TKA (cemented, hybrid, uncemented) for OA, adult patients (aged ≥ 18 years)	Preoperative anticoagulants (specifically, a direct oral anticoagulant, warfarin, or dual antiplatelet therapy) or those with a medical contraindication (allergy or bleeding disorder precluding anticoagulation)	Aspirin 100 mg/day orally or Enoxaparin 40 mg/day subcutaneously beginning within 24 h postoperatively, for 35 days after THA, 14 days after TKA. All patients received intraoperative and postoperative intermittent pneumatic compression calf devices until mobile, received compression stockings, and were offered mobilization on day 0 or day 1 postoperatively.	Symptomatic VTE within 90 days of surgery	Joint‐related readmission, joint‐related reoperation, major bleeding events (those resulting in readmission, reoperation, or death), and mortality within 90 days, joint‐related reoperation within 6 months of surgery, and adherence rates
3	CRISTAL Study Group 2023	Registry‐nested, crossover cluster‐randomized controlled trial, multicenter (secondary analysis)	Australia	Aspirin (*n* = 14156), Enoxaparin (*n* = 9302)	Patients with fracture and non‐fracture diagnosis (OA, inflammatory, avascular necrosis, other) undergoing total (primary, partial, resurfacing, and revision hip) or knee arthroplasty (cemented, hybrid, uncemented), adult patients (aged ≥ 18 years)	Preoperative anticoagulants (specifically, a direct oral anticoagulant, warfarin, or dual antiplatelet therapy) or those with a medical contraindication (allergy or bleeding disorder precluding anticoagulation)	Aspirin 100 mg/day orally or Enoxaparin 40 mg/day subcutaneously beginning within 24 h postoperatively, for 35 days after THA, 14 days after TKA. All patients received intraoperative and postoperative intermittent pneumatic compression calf devices until mobile, received compression stockings, and were offered mobilization on day 0 or day 1 postoperatively	All‐cause mortality within 90 days of surgery	Joint‐related readmission, joint‐related reoperation, major bleeding events (those resulting in readmission, reoperation, or death), and mortality within 90 days, joint‐related reoperation within 6 months of surgery, and adherence rates
4	CRISTAL Study Group 2024	Registry‐nested, crossover cluster‐randomized controlled trial, multicenter (secondary analysis)	Australia	Aspirin (*n* = 6901), Enoxaparin (*n* = 4827)	Patients with fracture and non‐fracture diagnosis (OA, inflammatory, Avascular necrosis, other) undergoing total, partial, resurfacing, and revision hip or knee arthroplasty (cemented, hybrid, uncemented), adult patients (aged ≥ 18 years)	Preoperative anticoagulants (specifically, a direct oral anticoagulant, warfarin, or dual antiplatelet therapy) or those with a medical contraindication (allergy or bleeding disorder precluding anticoagulation)	Aspirin 100 mg/day orally or Enoxaparin 40 mg/day subcutaneously beginning within 24 h postoperatively, for 35 days after THA, 14 days after TKA. All patients received intraoperative and postoperative intermittent pneumatic compression calf devices until mobile, received compression stockings, and were offered mobilization on day 0 or day 1 postoperatively	Symptomatic VTE within 90 days of surgery	Joint‐related readmission, joint‐related reoperation, major bleeding events (those resulting in readmission, reoperation, or death) and mortality within 90 days, joint‐related reoperation within 6 months of surgery, and adherence rates
5	Westrich et al., 2006	Randomized controlled trial, single center	USA	Aspirin (*n* = 129), Enoxaparin (*n* = 135)	Unilateral TKA, aged 38–86 years	Allergies to aspirin; congenital or acquired bleeding disorders; active ulcerative or angiodysplastic gastrointestinal disease; multiple myeloma or other paraproteinemias, pheochromocytoma, hyperthyroidism, impaired renal function, or known hepatic disease; past medical history of stroke; recent brain, spinal, or ophthalmologic surgery; hypersensitivity to enoxaparin sodium; cardiac complications; severe peripheral vascular diseases; chronic heart failure; severe varicose veins; or history of DVT and/or pulmonary embolism (PE)	All goups received spinal epidural anesthesia (SEA), pneumatic compresion device (VenaFlow calf). Enoxaparin was initiated 2 h after catheter removal (48 h postoperatively), 30 mg twice daily until discharge than 40 mg once daily for 3 weeks. Aspirin 325 mg twice daily started on the night after surgery and continued for 4 weeks	DVT during Doppler ultrasound screening on postoperative days 3 to 5 and 4 to 6 weeks postoperatively	Blood transfusion rate, tourniquet time, intraoperative blood loss, postoperative blood loss
6	Zhou et al., 2023	Randomized controlled trial, single center	China	Aspirin (*n* = 60), Dalteparin (*n* = 60), Rivaroxaban (*n* = 60)	Patients with OA who underwent primary unilateral TKA, aged 55–80 years	Secondary osteoarthritis, such as post‐traumatic arthritis, rheumatoid arthritis, and gouty arthritis, those with systemic or local infection; those with blood system diseases; those with previous or current use of antithrombotic drugs; those with a previous history of thrombosis or thrombosis discovered on color Doppler ultrasound of both lower extremities; those developing high‐risk cardiovascular disease (CVD) with thromboses, including cerebral infarction, myocardial infarction, atrial fibrillation, heart failure and post‐stenting; those taking nonsteroidal anti‐inflammatory drugs (NSAIDs); and those with a history of epilepsy or severe liver and kidney insufficiency	Intraoperative drip of 60 mg/kg Tranexamic acid. Then three groups received oral rivaroxaban at 10 mg, subcutaneous Dalteparin at 2500 IU, and oral aspirin at 100 mg. All treatments were given once a day for 30 days at 12 h postoperatively.	Posttreatment drainage volume and thrombotic complication rate within 90 days	Hematologic parameters on days 1, 3, and 5 after treatment, blood transfusion rate, total blood loss, intraoperative blood loss, and bleeding complication rate.
7	Zou et al., 2014	randomized, double‐blind, controlled trial, single center	China	Aspirin (*n* = 110), Enoxaparin (*n* = 112), Rivaroxaban (*n* = 102)	Patients with OA who underwent primary unilateral TKA (cemented), were DTV‐negative according to the preoperative color Doppler ultrasonography on the deep veins of both lower extremities, aged 47–82 years	History of hemorrhagic disease or a bleeding tendency during the preoperative coagulation test, had a medical history of VTE, were infused with over 2000 mL of fluids 24 h after surgery, underwent knee arthroplasty, or used a combination of other drugs that might impact the findings	12 h after the operation three groups received oral rivaroxaban 10 mg/day, enoxaparin sc 4000 AxaIU (0.4 mL/day) oral aspirin at a dose of 100 mg/day. All of the groups were treated for 14 days. Ankle pump exercises began 6 h after the surgery. A pressure dressing was applied to the affected extremities with elastic bandages, and mobilization	Routine blood tests performed 3 days, 7 days and 14 days after surgery	DVT within 14 and 28 days after surgery, wound healing and wound complications within 28 days after surgery

Abbreviations: OA, osteoarthritis; THA, total hip arthroplasty; TKA, total knee arthroplasty.

Bukhari et al. (2023) performed a small single‐center RCT in Pakistan and reported no difference in aspirin (75 mg/day orally) or enoxaparin (0,5 mg/kg s.c.) given for 14 days in the VTE rate within 14 days of follow‐up after THA [19]. No other outcomes or longer follow‐up have been reported. Two small single‐center RCTs from China evaluated three thromboprophylaxis approaches in patients with osteoarthritis who underwent primary unilateral TKA; aspirin (100 mg/day orally), LMWH [enoxaparin s.c. 4000 AxaIU (0,4 mL/day) and dalteparin at 2500 IU] and rivaroxaban (10 mg/day orally), all given 14 days and 30 days in Zou et al. (2014) [20] and Zhou et al. (2023) [21], respectively. Zou et al. (2014) reported no significant difference between aspirin and enoxaparin when given for 14 days in DVT, bleeding events, and wound complications within 30 days of follow‐up [20]. Zhou et al. (2023) reported no significant difference between aspirin and dalteparin when given for 30 days in DVT, PE, and bleeding event rates within 90 days of follow‐up. However, the authors reported a higher blood transfusion rate in the dalteparin group [21]. Westrich et al. (2006) also performed a single RCT in the United States with patients undergoing unilateral TKA [22]. Enoxaparin was initiated 2 h after catheter removal (48 h postoperatively), 30 mg twice daily until discharge, then 40 mg once daily for 3 weeks. Aspirin 325 mg twice daily was started on the night after surgery and continued for 4 weeks. The authors reported no statistical difference in DVT events within 4 to 6 weeks postoperatively in follow‐up, but there was a trend toward a lower DVT rate with enoxaparin. Other important outcomes were recorded only in the aspirin group, where 1 patient experienced an internal bleeding complication and 1 patient was confirmed to have a PE.

The CRISTAL trial (2022) was a registry‐nested [the Australian Orthopedic Association National Joint Replacement Registry (AOANJRR)], crossover cluster‐RCT, multicenter study, performed across 31 hospitals in Australia [23]. CRISTAL included primary elective total unilateral or bilateral THA or TKA (cemented, hybrid, or uncemented) for osteoarthritis. Participants were eligible to participate if they had received single antiplatelet therapy (e.g., aspirin) for preexisting medical conditions. In that case, the aspirin was continued preoperatively and postoperatively per local hospital practice and beyond the 14‐ or 35‐day prophylaxis period as indicated by a patient's condition. Participants were ineligible only if receiving preoperative anticoagulants (e.g., a direct oral anticoagulant, warfarin, or dual antiplatelet therapy) or those with a medical contraindication (allergy or bleeding disorder precluding anticoagulation). TKA procedures were more common (62%) than THA procedures (38%), particularly unilateral TKA and THA procedures (approximately 90%). Patients were given aspirin (100 mg/day orally) or enoxaparin (40 mg/day s.c.) starting within 24 h postoperatively, administered for 35 days after THA and 14 days after TKA, and were followed up within 90 days. All patients received intraoperative and postoperative intermittent pneumatic compression calf devices until mobile, wore compression stockings, and were encouraged to mobilize on day 0 or day 1 postoperatively. Crossover occurred after the patient enrollment target had been met for the first group. The trial enrolled 9711 of the planned 15 562 patients since it stopped early after an interim analysis determined that the stopping rule was met. Symptomatic VTE was significantly higher with aspirin (3,45%) than enoxaparin (1,82%), *p* = 0,007. Any DVT was also higher with aspirin (2,58% vs. 1,32%), which is affected mainly by the difference in DDVT (2,38% vs. 1,19%) and not PDVT (0,22% vs. 0,16%). Aspirin was non‐inferior to enoxaparin in terms of all‐cause mortality rate, but although statistically insignificant, a trend toward a higher PE rate might be present (1,07% vs. 0,55%). There was no difference in the adherence rate between aspirin (85%) and enoxaparin (86%).

The secondary analysis of the CRISTAL trial (2023) provided more useful data on the thromboprophylaxis effect in orthopedic patients [24]. The CRISTAL trial [23] included only patients with osteoarthritis undergoing primary total arthroplasty, while the secondary analysis [20] expanded the inclusion criteria to involve more patients (23458). The analysis included patients undergoing any hip or knee arthroplasty procedure [including total (81%), partial (10%), resurfacing (0,5%), and revision (8%)] for any indication at participating hospitals during the course of the study [24]. Besides osteoarthritis (78%), they included non‐osteoarthritis diagnoses [inflammation (1%), avascular necrosis (2%), fracture (11%), and other causes (9%)] as well. Aspirin was again confirmed to be non‐inferior to enoxaparin in terms of all‐cause mortality rate [24]. However, mortality was significantly higher in fracture cases (13,07% vs. 10,95%) compared to non‐fracture cases (0,49% vs. 0,41%), regardless of thromboprophylaxis type.

Another secondary analysis of the CRISTAL trial (2024) [25] reported on the postoperative occurrence of VTE (including individual components), bleeding, readmission, and reoperation in 11 728 participants with expanded inclusion criteria. It involved patients with fracture and non‐fracture diagnoses undergoing total, partial, resurfacing, and revision hip or knee arthroplasty. Primary total hip and knee arthroplasty procedures were the most common (approximately 93%). The secondary analysis of the CRISTAL trial (2024) [25] further reinforced the findings of the original CRISTAL trial [23]. Aspirin showed a statistically significant higher rate of any symptomatic VTE (3,27% vs. 1,76%), any DVT (2,45% vs. 1,24%), with the most pronounced difference in DDVT (2,25% vs. 0,83%), as well as an increased reoperation rate (2,48% vs. 2,26%) within 90 days. This difference in increased reoperation rate was not seen within 6 months (4,26% vs. 4,05%) [25].

### 3.2 Implications on Budget

We used the CRISTAL trial (2022) [23] and the secondary analysis of the CRISTAL trial results (2024) [25] for the simplified budget impact analysis. The CRISTAL trial is a large multicenter RCT with documented various efficacy and safety outcomes. Other RCTs were excluded due to their smaller sample sizes, single‐center nature, and potential biases [19, 20, 21, 22]. In the secondary analysis of the CRISTAL trial (2024) [25], secondary outcomes were analyzed without a multiplicity adjustment, and the results may be due to chance, although subpopulations were likely balanced due to the large trial population (11728). We used the following variables for the budget impact analysis: VTE symptomatic, major bleeding, and reoperation outcomes percentage (%), dosing [two times a day (b.i.d.) and once a day (q.d.)] and duration of aspirin and enoxaparin therapy, and ASP drug pricing (Table 3) [16, 23, 25]. We excluded all‐cause mortality and readmission variables for the analysis since there was no significant difference nor trend in favor of any thromboprophylaxis strategies for these outcomes. For THA and TKA procedure volume in patients with osteoarthritis, we used annual estimates of 635,000 and 1,257,800 cases, respectively [13]. Although the secondary analysis of the CRISTAL trial (2024) [25] involves patients undergoing total, partial, resurfacing, and revision hip or knee arthroplasty procedures, THA and TKA procedure volume was used for calculations since approximately 93% of all hip and knee arthroplasty procedures are primary total procedures. The price per VTE event including complications was $23000, and per major bleeding event was $9824 [14, 15]. The price per revision TKA in high‐income countries for the middle‐priced revision (1‐ or 2‐stage septic revision without re‐revision) was $38109 [17]. The price for revision THA for the middle‐priced revision of septic revision was $17696 [18]. Both CRISTAL publications report on joint‐related (hip and knee arthroplasty) reoperation rates. Reoperation rates range from 1% to 1,6% in the first 3 months and first year after TKA and from 1,6% to 5,4% at ≤ 5‐year post THA in the literature [26, 27]. Therefore, we approximated that TKA and THA occurred in CRISTAL with roughly the same incidence (ranging from 2% to 2,5%) in the first 3 months and were used as such in calculations. The approximated yearly incidence of VTE, major bleeding, and reoperation events was calculated using variables (%) from the CRISTAL trial. Using the CRISTAL trial (2022) [23] results, we calculated annual costs of $2,838,401,577 and $ 2,727,970,336 for USA healthcare stakeholders for aspirin and enoxaparin thromboprophylaxis in patients with osteoarthritis undergoing TKA and THA, respectively (Table 3). From a single hospital DTC perspective, assuming 1000 combined TKA and THA procedures annually (distribution 66% vs. 34%), aspirin and enoxaparin costs were $1,497,639 and $ 1,441,189, respectively. The secondary analysis of the CRISTAL trial results (2024) [25] demonstrated similar costs for aspirin and enoxaparin thromboprophylaxis, $2,956,085,282 and $ 2,920,172,823, respectively. Assuming 1000 combined TKA and THA procedures annually (66% vs. 34%), aspirin and enoxaparin costs were $1,559,508 and $ 1,542,432, respectively. It appears that aspirin's lower effectiveness on symptomatic VTE prevention also affected the budget. Thromboprophylaxis with enoxaparin appears to produce probably between $35,912,459 and $ 110,431,241 of yearly savings or $17,075 and $ 56,450000 combined TKA and THA (660 TKA and 340 THA) procedures (Table 3).

## 4 Discussion

Aspirin is non‐inferior to LMWH in terms of all‐cause mortality but is inferior in preventing symptomatic VTE when used as a sole thromboprophylactic agent immediately postoperatively in patients undergoing hip or knee arthroplasty (Table 2). Our findings align with a recent systematic review and meta‐analysis on TKA [28]. Although these results should be interpreted cautiously due to methodological concerns arising from high variability in available RCTs [29]. The Salman et al. meta‐analysis on patients undergoing hip or knee arthroplasty used different inclusion criteria compared to ours. The authors also included RCTs with alternative forms of prophylaxis for the immediate postoperative period prior to aspirin prescription (e.g., the EPCAT trial [10]). Despite differences in inclusion criteria, their findings align with ours. There was no difference in terms of all‐cause mortality, but the deleterious effect of aspirin on PE risk was evident [30]. Although meta‐analyses are compelling, previous reviews and meta‐analyses have noted variability and low quality of evidence in RCTs on this topic [31]. Therefore, our research may offer more compelling insights than a meta‐analysis, given the significant differences across RCTs in terms of population, interventions (concomitant medications), dosing, outcomes, and timing of assessments (Table 1) [19, 20, 21, 22, 23, 24, 25]. We have also performed a simplified budget impact analysis from a healthcare stakeholder or hospital DTC perspective using available evidence. Thromboprophylaxis with LMWH (particularly enoxaparin) appears to yield yearly savings between $35 912 459 and $110 431 241 for healthcare stakeholders compared to aspirin (Table 3).

**TABLE 2:** Overview of outcomes from eligible RCTs that compared aspirin versus LMWH for thromboprophylaxis following hip or knee arthroplasty.

Outcome/Variable	Bukhari et al., 2023 (No./%)		CRISTAL study group 2022 (No./%)		CRISTAL study group secondary analysis 2023 (No./%)		CRISTAL study group secondary analysis 2024 (No./%)		Westrich et al., 2006 (No./%)				Zhou et al., 2023 (No./%)		Zou et al., 2014 (No./%)	
	Aspirin	Enoxaparin	Aspirin	Enoxaparin	Aspirin	Enoxaparin	Aspirin	Enoxaparin	Aspirin	Enoxaparin	Aspirin	Enoxaparin	Aspirin	Dalteparin	Aspirin	Enoxaparin
Number of patients	37	37	5416	3787	14154	9299	6901	4827	136	139	136	139	60	60	110	112
Median age	48	51	67	68	69	70	68	69	69	69	69	69	66	64	63	66
Population	Patients undergoing THA or hemiarthroplasty		Patients with osteoarthritis undergoing THA or TKA		Patients with fracture and non‐fracture diagnosis undergoing total, partial, resurfacing, and revision hip or knee arthroplasty		Patients with fracture and non‐fracture diagnosis undergoing total, partial, resurfacing, and revision hip or knee arthroplasty		Patients undergoing unilateral TKA				Patients with osteoarthritis undergoing primary unilateral TKA		Patients with osteoarthritis undergoing unilateral TKA	
Dosing	75 mg qd	0,5 mg/kg qd	100 mg qd	40 mg qd	100 mg qd	40 mg qd	100 mg qd	40 mg qd	325 mg bid	30 mg bid till discharge than 40 mg qd	325 mg bid	30 mg bid till discharge than 40 mg qd	100 mg qd	2500 IU qd	100 mg qd	4000 IU qd
Duration of thromboprophylaxis (median)	14		35 THA, 14 TKA		35 THA, 14 TKA		35 THA, 14 TKA		28				30		14	
All‐cause mortality			0.07%	0.05%	1.67%	1.53%	0.12%	0.08%								
Nonfracture diagnosis					0.49%	0.41%										
Fracture diagnosis					13.07%	10.95%										
VTE symptomatic	5.41%	10.81%	3.45%	1.82%			3.27%	1.76%								
PE			1.07%	0.55%			1.00%	0.56%			0.74%		0.00%	0.00%	0.00%	0.00%
DVT any			2.58%	1.32%			2.45%	1.24%	14.00%	12.60%	5.43%	2.02%			18.18%	13.39%
DVT asymptomatic															16.36%	12.50%
DVT symptomatic															1.82%	0.89%
PDVT events (proximal—above‐knee)			0.22%	0.16%			0.20%	0.21%	0.01%	0.04%	1.09%	0.00%	0.00%	1.70%		
DDVT events (distal—below‐knee)			2.38%	1.19%			2.25%	0.83%	11.76%	8.63%	4.35%	2.02%	11.70%	6.70%		
Major bleeding			0.31%	0.40%			0.29%	0.52%			0.74%		8.30%	10.00%	0.00%	0.00%
Readmission			2.41%	2.25%			2.85%	2.76%								
Reoperation			2.14%	1.93%			2.48%	2.26%								
Wound complications															1.82%	2.67%
Outcomes assessment	Within 14 days		Within 90 days		Within 90 days		Within 90 days		At discharge		4–6 weeks after discharge		Within 90 days		Within 30 days	

*Note:* Statistically significant difference (*p* < 0.05).

Abbreviations: DDVT, distal to the popliteal vein deep vein thrombosis; DVT, deep vein thrombosis; OA, osteoarthritis; PDVT, proximal to the popliteal vein deep vein thrombosis; PE, pulmonary embolism; THA, total hip arthroplasty; TKA, total knee arthroplasty; VTE, venous thromboembolism.

**TABLE 3:** Budget impact model for aspirin versus LMWH for thromboprophylaxis following hip or knee arthroplasty.

	Annual cost for overall TKA and THA procedures in the United States				Annual cost for 1000 TKA and THA procedures			
	CRISTAL study group 2022 (No./%)		CRISTAL study group secondary analysis 2024 (No./%)		CRISTAL study group 2022 (No./%)		CRISTAL study group secondary analysis 2024 (No./%)	
Outcome/variable	Aspirin	Enoxaparin	Aspirin	Enoxaparin	Aspirin	Enoxaparin	Aspirin	Enoxaparin
VTE symptomatic	3.45%	1.82%	3.27%	1.76%	3.45%	1.82%	3.27%	1.76%
Major bleeding	0.31%	0.40%	0.29%	0.52%	0.31%	0.40%	0.29%	0.52%
Reoperation (TKA and THA)	2.14%	1.93%	2.48%	2.26%	2.14%	1.93%	2.48%	2.26%
% TKA procedures	62.03%	61.89%	61.45%	60.26%	62.03%	61.89%	61.45%	60.26%
% THA procedures	37.97%	38.11%	38.55%	39.74%	37.97%	38.11%	38.55%	39.74%
Dosing	100 mg qd	40 mg qd	100 mg qd	40 mg qd	100 mg qd	40 mg qd	100 mg qd	40 mg qd
Duration of thromboprophylaxis (knee arthroplasty)	14				14			
Duration of thromboprophylaxis (hip arthroplasty)	35				35			
Cost per tablet/vial	$0.12	$18.00	$0.12	$18.00	$0.12	$18.00	$0.12	$18.00
Total cost of thromboprophylaxis/patient TKA	$1.68	$252.00	$1.68	$252.00	$1.68	$252.00	$1.68	$252.00
Total cost of thromboprophylaxis/patient THA	$4.20	$630.00	$4.20	$630.00	$4.20	$630.00	$4.20	$630.00
Cost of VTE event/patient	$23000.00				$23000.00			
Cost of a bleeding event/patient	$9824.00				$9824.00			
Mean cost of reoperation/patient for TKA	$38109.00				$38109.00			
Mean cost of reoperation/patient for THA	$17969.00				$17969.00			
Yearly incidence of TKA	1 257 800				660			
Yearly incidence of THA	635 000				340			
Approximate yearly incidence of VTE	65353	34487	61987	33331	35	18	33	18
Approximate yearly incidence of Major bleeding	5958	7513	5502	9822	3	4	3	5
Approximate yearly incidence of Reoperation for TKA	26959	24246	31190	28403	14	13	16	15
Approximate yearly incidence of Reoperation for THA	13610	12241	15746	14339	7	7	8	8
All cost (thromboprophylaxis, VTE, major bleeding, reoperation)	$2838401577	$2727970336	$2956085282	$2920172823	$1497639	$1441189	$1559508	$1542432
**Δ**	$110 431 241		$35 912 459		$56 450		$17 075	

While aspirin and LMWH have both been examined as possible methods for preventing VTE in previous RCTs [21, 22], it is the CRISTAL trial [23] that provides the most compelling evidence thus far. Thus, it serves as our main point of reference. Other studies that were identified during our systematic review appeared to be rapid inquiries characterized by low‐quality reporting, different methodologies, or studied populations beyond our main interest in arthroplasty patients (Table 1) [19, 20, 21, 22]. Therefore, the CRISTAL trial [23], with its secondary analyses [24, 25], has primarily been used to determine valid and clinically relevant conclusions in this research.

The CRISTAL trial reported higher overall VTE rates with aspirin than with LMWH; these differences were primarily driven by an increased incidence of DDVT. Unlike PDVT, DDVT is generally considered less clinically significant, often not requiring anticoagulation, as the benefits are offset by an increased bleeding risk. Additionally, non‐trauma patients are less likely to experience clot propagation and embolization [32, 33]. Aspirin demonstrated higher rates of symptomatic VTE compared to LMWH with incidences of 3,45% vs. 1,82% and 3,27% vs. 1,76% in the CRISTAL trial (2022) [23] and the secondary analysis of the CRISTAL trial (2024) [25], respectively. As such, the increased DDVT rates with aspirin may not necessitate intervention or alter patient prognosis significantly. Furthermore, this distinction underscores that despite aspirin's higher overall VTE rate, its non‐inferiority to LMWH in terms of all‐cause mortality remains a crucial finding for clinical decision‐making. The CRISTAL trial [23] also found a tendency (statistically insignificant but numerically higher) toward a greater risk of PE in the aspirin group (1,07%) compared to the LMWH group (0,55%). PE is much more concerning for patient outcomes and is associated with poorer survival rates than DVT alone [34]. Given the low overall incidence of PE in both groups, the clinical significance of this finding is uncertain. Nevertheless, these data highlight the need for continued investigation into PE risk when using aspirin as the sole agent for postoperative thromboprophylaxis in arthroplasty patients.

All‐cause mortality is much more pronounced despite aspirin or enoxaparin thromboprophylaxis in fracture indications (13,07% vs. 10,95%) than in non‐fracture (0,49% vs. 0,41%) indications [24]. This difference is likely related to the frailty of patients and the independent risk that fractures pose for poor outcomes in the elderly. Frailty in older surgical patients is a strong predictor for postoperative mortality and complications, irrespective of the type of surgery [35]. Indeed, the Hip Fracture Program for Elders documented a 1‐year mortality rate of 21,2% in patients ≥ 60 years of age with hip fractures. Age, male gender, low Parker mobility score, and a Charlson score of 4 or greater have all been positively correlated with mortality [36]. The CRISTAL trial (2022) [23] included solely patients with osteoarthritis (median age 68 years) with mortality rate (0,07% vs. 0,05%) while the secondary analysis of the CRISTAL trial (2023) [24] (median age 70 years) included 78% of patients with osteoarthritis, while only 11% of patients with fractures, and mortality rate (1,67% vs. 1,53%). Elderly patients with fractures undergoing THA and TKA likely contribute to this elevated mortality rate.

The CRISTAL trial (2022) [23] is a registry‐nested, crossover cluster RCT that represents everyday clinical practice [37] (included patients within a registry), but the data on adherence rates between aspirin and enoxaparin (85% vs. 86%) should be interpreted with caution. The adherence rate was affected mainly (82%) by inpatient time rather than post‐discharge (18%) time (1005 patients audited for inpatient and 178 patients audited for post‐discharge drug adherence). Therefore, adherence to aspirin or enoxaparin post‐discharge, when patients are not monitored by healthcare professionals, is less known [23]. It is unreported in the CRISTAL trial (2022) [23] what the median inpatient stay was. We can assume from previous publications that discharge is within a few days, where Kester et al. also reported that for hip and knee arthroplasty, the 57,9% and 38,3% of VTE events occurred after discharge, respectively [38]. Indeed, in the CRISTAL trial (2022) [23], the median time to diagnosis of symptomatic VTE was 7,5 days (IQR, 5–19 days) and 12 days (IQR, 7–25 days) in aspirin and enoxaparin groups, respectively. Other RCTs involving trauma patients report that aspirin tablets are more convenient than enoxaparin vials, increasing patient satisfaction [39, 40]. Consequently, this could increase patient adherence, which leads to achieving pharmacotherapy goals. Adherence is of great importance since trial patients are more motivated and followed closely when compared to everyday clinical practice [41]. We can conclude that drug adherence post‐discharge is of great importance, and aspirin is more convenient for patients than LMWH.

When balancing quality, safety, and economic factors, LMWH likely emerges as the more cost‐effective option. The recent high‐quality cost‐effectiveness analysis (CEA) [42] of the CRISTAL trial (2022) [23] reported with 60% confidence that the incremental cost per quality‐adjusted life‐year (QALY) does not exceed the willingness‐to‐pay threshold of AUD$70 000. The CEA was conducted from an Australian healthcare stakeholder perspective. Our simplified budget impact analysis, based on the published data from the CRISTAL trial (2022) [23] and the secondary analysis of the CRISTAL trial (2024) [25], indicates that although aspirin is significantly cheaper per tablet ($0,10–$0,12) compared to enoxaparin per vial ($12,80–$18,00), aspirin is likely a more expensive thromboprophylactic intervention overall. The yearly costs for aspirin and enoxaparin thromboprophylaxis in patients with osteoarthritis undergoing THA and TKA were $2838401577 versus $2,727,970,336 and $ 2,956,085,282 versus $2920172823, respectively, for U.S. healthcare stakeholders (Table 3). Aspirin's relatively poorer outcomes, including higher VTE and reoperation rates, contributed to the increased budget impact. Thus, enoxaparin thromboprophylaxis appears to result in yearly savings for healthcare stakeholders, ranging between $35 912 459 and $110 431 241 (Table 3). Assuming a total of 1000 combined TKA and THA procedures (660 TKA and 340 THA), enoxaparin results in yearly savings ranging between $17 075 and $56 450 (Table 3).

Our research has several limitations. First, our conclusions are primarily based on the CRISTAL trial [23] and its secondary analyses [24, 25], which provide somewhat limited evidence. However, as we have documented, other RCTs [19, 20, 21, 22] fulfilling inclusion criteria are of lower quality. They include limitations such as short follow‐up periods, small sample sizes, single‐center RCTs, and comparisons involving multiple interventions rather than a direct aspirin versus LMWH comparison, as these factors reduce their statistical value. Despite limitations within the CRISTAL trial [23] itself, such as early termination due to aspirin's inferiority regarding the primary outcome (VTE) in the interim analysis, it still offers valuable insights for clinical practice. The trial's design as a registry‐nested, crossover cluster RCT closely reflects everyday clinical practice [23, 37]. Furthermore, it is a multicenter trial with a large trial population that strengthens the credibility of evidence, even with some potential biases (e.g., crossover, stopping early, lack of adjustment for multiplicity, etc.). Also, its secondary analyses [24, 25] expanded the inclusion criteria and consequently incorporated even more patients. Despite some bias factors (e.g., lack of adjustment for multiplicity), they produced similar results as the CRISTAL trial itself, further fortifying the results of the CRISTAL trial (2022) [23]. Aspirin and LMWH are affordable, long‐established thromboprophylactic agents on the drug market and available as generics. We should not expect large industry‐sponsored RCTs on this topic in the near future. Therefore, the CRISTAL trial publications [23, 24, 25, 42] could be the best available evidence for the long term. Second, meta‐analyses provide the highest quality of evidence to answer clinical questions. However, as we have argued, due to the high variability of RCTs on this topic, meta‐analyses are probably not the best method of analysis. Therefore, we chose to analyze each included RCT separately, which we consider a strength of our research. Third, we used the incidence of outcomes of interest from a registry‐nested, crossover cluster RCT rather than a more traditional RCT. As we have stated earlier, the CRISTAL trial [23] provides pragmatic insights into important clinical questions and provides the necessary data for our budget impact analysis.

Despite adhering to the methodology guidelines [43] our budget impact analysis has several limitations. We have used a simplified economic model rather than a complex linear regression model, which requires extensive RCT data along with statistical and programming software for analysis. We opted for a more simplistic approach due to data availability in the literature and the fact that some healthcare stakeholders and, more often, hospital DTCs do not perform CEA that can be readily used for a more complex budget impact analysis. Simplified budget impact analyses, like ours, are still used in everyday practice. There are several reasons for this, such as time constraints for healthcare professionals who serve on DTCs, restricted access to comprehensive data required to perform CEA, and limited access to statistical and programming software. Therefore, we chose to perform a simplified budget impact analysis alongside a systematic review to provide additional insights for clinicians on this topic. We used outcomes and variables that may impact the costs: VTE, major bleeding, reoperation, and thromboprophylaxis cost. Incidence and cost data were derived from the CRISTAL trial publications [23, 25] and the literature [13, 14, 15, 16, 17, 18]. Another limitation is that we did not perform a sensitivity analysis for the budget impact analysis. Regarding healthcare professional costs, we assumed a similar length of stay in both groups and therefore comparable healthcare professional costs. Outpatient daily self‐injection of LMWH by the patients or their families is a common practice, and self‐injection is refused only by a small percentage of patients (7,7%) [44]. Therefore, we assumed that enoxaparin is self‐administered and not given by a healthcare professional, consequently not affecting the cost. We did not include all‐cause mortality and readmission costs in our analysis, which may slightly underestimate the total yearly costs of THA and TKA procedures in the United States. However, since there is no statistically significant difference or trend between aspirin and LMWH for these outcomes, and all‐cause mortality rates are negligible, including these costs would not affect the absolute cost difference between thromboprophylaxis interventions. For the secondary analysis of the CRISTAL trial (2024) [25], we used THA and TKA procedure volumes to estimate the total cost of knee and hip arthroplasty, rather than including all types of knee and hip surgeries. This approach was chosen due to limited cost data available on these procedures, and because approximately 93% of the hip and knee surgeries in the secondary analysis of the CRISTAL trial (2024) [25] were primary total arthroplasties. We also approximated the yearly cost of aspirin and enoxaparin thromboprophylaxis on a much smaller scale (1000 combined TKA and THA procedures) to provide a more representative estimate for single‐hospital calculations. Despite these limitations, our more simplistic approach to budget impact analysis produced similar conclusions in favor of enoxaparin, as demonstrated in the higher quality CEA of the CRISTAL trial [42].

## 5 Conclusion

Enoxaparin has been shown to be non‐inferior to aspirin in terms of all‐cause mortality and superior in reducing VTE rates, particularly DDVT, when used as a sole thromboprophylactic agent immediately after hip or knee arthroplasty. The observed higher incidence of PE with aspirin raises concerns and warrants further investigation. Although aspirin demonstrated a trend toward fewer major bleeding events and is more convenient to use than LMWH, these advantages did not result in lower overall thromboprophylaxis costs. In fact, enoxaparin may generate annual savings of $35 912 459 to $110 431 241 for healthcare stakeholders in the United States when compared to aspirin in patients undergoing hip or knee arthroplasty. Further research from the academic community is needed to fully address this clinical question.

## Author Contributions

E.J. and M.L. designed the study. E.J. performed the budget‐analytical model. E.J. and A.B. analyzed the data, assembled, and led the drafting of the manuscript. A.F. completed the search strategy. G.H., P.J., M.S., A.F., and I.K. critically reviewed the manuscript. All authors have interpreted the results and have approved the final manuscript.

## Conflicts of Interest

The authors declare no conflicts of interest.

## Data Availability

The data that support the findings of this study are available upon reasonable request sent to the corresponding author.
